# Response to “Methodological issues in the designing and reporting of frequentist and Bayesian meta‐analysis to assess COVID‐19 outcomes among PLHIV with various comorbidities” by Ram Bajpai, Vivek Verma and Gyan Prakash Singh

**DOI:** 10.1002/jia2.25947

**Published:** 2022-06-09

**Authors:** Haoyi Wang, Kai J. Jonas

**Affiliations:** ^1^ Department of Work and Social Psychology Maastricht University Maastricht The Netherlands

**Keywords:** comorbidities, corona virus, COVID‐19, HIV, Key and vulnerable populations, meta‐analysis

1

Our colleagues Bajpai et al. have submitted a Letter to the Editor regarding our recently published meta‐analysis “The likelihood of severe COVID‐19 outcomes among PLHIV with various comorbidities: a comparative frequentist and Bayesian meta‐analysis approach [1].” We appreciate their feedback and request for clarification and would like to respond to their letter.

First, we have indeed used the Preferred Reporting Items for Systematic reviews and Meta‐Analyses 2020 in our flow diagram. Unfortunately, we cited the wrong resource for this. We apologize for this error, yet we deem the impact to be rather small as the flow diagram is correct. We did not register the meta‐analysis with PROSPERO as the registration procedure currently takes more than 4 months. The urgency and timely relevance of the analysis did not allow for a registration, from our point of view. In times of crisis, a value weighing is necessary between adhering to time‐intensive procedures or contribute to directly relevant care provision.

Second, the authors suggest that there is missing information about the model selection (i.e. fixed or random). We disagree with this view. A random‐effects model assumes that each study estimates a different underlying true effect, and these effects have a distribution (usually a normal distribution). A fixed‐effects model should be used only if it is reasonable to assume that all studies share the same, common effect [2]. If it is not reasonable to assume that there is one common effect size, then the random‐effects model should be used. If the studies are heterogeneous from a clinical and methodological point of view, it is unreasonable to assume that they share a common effect [2]. In our study, given the heterogeneous studies included as well as their purposes (different comorbidities, different settings and different study designs), it is not reasonable to use fixed‐effect model. We believe it was not necessary to further motivate our selection of a random effects model as the default, and we consider this such a claim irrelevant for this paper, as this is not a methods paper.

Third, the authors ask us to present the results with the prediction interval. This is a recent recommendation that is not yet standard practice. Please find the prediction interval reported in Table 1.

**Table 1:** Model summary with classical frequentist prediction interval

								Classical frequentist approach			Bayesian approach with half normal distribution prior^b^		
Comorbidity	Study	Year	Country	Sample size	COVID‐19 outcome	Log odds ratio (95% CI)	σ_i_	Pooled effect (95% CI)	τ (95% CI)	Prediction interval	Fixed effect (95% CrI)	Random effect τ (95% CrI)	Predictive distribution (95% CrI)
Diabetes	Bhaskaran et al. [11]	2021	UK	27,480	Mortality	3.63 (2.15–5.11)	0.57	1.90 (1.11–2.69)	0.82 (0.12–2.42)	(0.11–3.69)	1.92 (1.22–2.57)	0.54 (0–1.05)	1.94 (0.41–3.37)
	Boulle et al. [12]	2020	South Africa	3978	Mortality	2.26 (1.88–2.64)	0.04						
	Ceballos et al. [13]	2020	Chile	36	Mortality	2.2 (–0.65 to 5.04)	2.11						
	Dandachi et al. [14]	2020	US	286	Hospitalization^a^	1.14 (0.31–1.96)	0.18						
	Etienne et al. [15]	2020	France	54	Severe and critical	3.91 (0.84–6.98)	2.45						
	Isernia et al. [16]	2020	France	30	Hospitalization	2.93 (–0.16 to 6.03)	2.49						
	Meyerowitz et al. [17]	2020	US	36	Hospitalization	0.73 (–1.21 to 2.68)	0.98						
	Pujari et al. [18]	2021	India	86	Severe and critical	1.53 (–0.11 to 3.17)	0.70						
	Vizcarra et al. [19]	2020	Spain	51	Severe and critical	–0.47 (–2.86 to 1.92)	1.48						
Hypertension	Bhaskaran et al. [11]	2021	UK	27,480	Mortality	3.02 (1.55–4.50)	0.57	1.42 (0.75–2.10)	0.56 (0–1.84)	(0.13–2.71)	1.38 (0.76–2.02)	0.41 (0–0.97)	1.37 (0.20–2.62)
	Ceballos et al. [13]	2020	Chile	36	Mortality	2.2 (–0.42 to 4.81)	1.78						
	Dandachi et al. [14]	2020	US	286	Hospitalization^a^	1.08 (0.36–1.80)	0.13						
	Etienne et al. [15]	2020	France	54	Severe and critical	1.86 (0.40–3.32)	0.55						
	Isernia et al. [16]	2020	France	30	Hospitalization	2.25 (–0.84 to 5.34)	2.49						
	Meyerowitz et al. [17]	2020	US	36	Hospitalization	0.04 (–1.73 to 1.81)	0.82						
	Pujari et al. [18]	2021	India	86	Severe and critical	1.56 (0.16–2.96)	0.51						
	Vizcarra et al. [19]	2020	Spain	51	Severe and critical	0.37 (–1.14 to 1.88)	0.59						
Cardiovascular disease	Ceballos et al. [13]	2020	Chile	36	Mortality	3.3 (0.23–6.36)	2.44	1.56 (0.64–2.48)	0.58 (0–2.82)	(0.08–3.04)	1.55 (0.69–2.41)	0.35 (0–0.94)	1.55 (0.20–2.89)
	Dandachi et al. [14]	2020	US	286	Hospitalization^a^	1.95 (0.81–3.10)	0.34						
	Etienne et al. [15]	2020	France	54	Severe and critical	1.61 (0.27–2.95)	0.47						
	Isernia et al. [16]	2020	France	30	Hospitalization	1.84 (–1.52 to 5.19)	2.94						
	Meyerowitz et al. [17]	2020	US	36	Hospitalization	2.4 (–0.78 to 5.57)	2.63						
	Vizcarra et al. [19]	2020	Spain	51	Severe and critical	–0.47 (–2.33 to 1.39)	0.90						
Respiratory disease	Ceballos et al. [13]	2020	Chile	36	Mortality	0.75 (–2.89 to 4.38)	3.44	1.30 (0.58–2.02)	0 (0–1.43)	(0.58–2.02)	1.23 (0.35–2.08)	0.28 (0–0.82)	1.24 (–0.04 to 2.42)
	Dandachi et al. [14]	2020	US	286	Hospitalization^a^	1.58 (0.67–2.50)	0.22						
	Etienne et al. [15]	2020	France	54	Severe and critical	1.2 (–0.88 to 3.29)	1.13						
	Isernia et al. [16]	2020	France	30	Hospitalization	0.29 (–1.13 to 5.71)	3.04						
	Meyerowitz et al. [17]	2020	US	36	Hospitalization	0.22 (–2.14 to 2.58)	1.45						
	Vizcarra et al. [19]	2020	Spain	51	Severe and critical	0.22 (–2.29 to 2.74)	1.65						
Chronic kidney disease	Bhaskaran et al. [11]	2021	UK	27,480	Death	3.74 (2.20–5.27)	0.61	2.20 (0.93–3.47)	1.02 (0–3.96)	(–0.19 to 4.58)	2.09 (1.15–3.07)	0.47 (0–1.08)	2.09 (0.54–3.72)
	Ceballos et al. [13]	2020	Chile	36	Mortality	2.2 (–0.65 to 5.04)	2.11						
	Dandachi et al. [14]	2020	US	286	Hospitalization^a^	1.58 (0.67–2.50)	0.22						
	Etienne et al. [15]	2020	France	54	Severe and critical	3.46 (0.33–6.60)	2.56						
	Meyerowitz et al. [17]	2020	US	36	Hospitalization	0.22 (–2.14 to 2.58)	1.45						

Note: Hospitalization includes hospitalized but not requiring supplemental oxygen, hospitalized but requiring supplemental oxygen, hospitalized with non‐invasive ventilation, hospitalized on invasive mechanical ventilation or ECMO and mortality; severe outcome was defined as fever or suspected respiratory infection plus respiratory rate greater than 30 breaths per min, oxygen saturation of 93% or less on room air, or acute severe respiratory distress (acute lung infiltrate in chest imaging and ratio of partial pressure of arterial oxygen to fractional concentration of oxygen in inspired air [PaO2/FiO2] of ≤300). Critically ill individuals were those with rapid disease progression and respiratory failure with need for mechanical ventilation or organ failure that needs monitoring in an intensive care unit. The scale on the effects is log odds ratio.

Abbreviations: 95% CI, 95% confidence interval; 95% CrI, 95% credible interval.

^a^ Did not report type of hospitalization.

^b^ Half‐normal distributions with scale 0.5.

Fourth, the authors believe that our publication bias assessment is prone to chance bias. We disagree with this view. Indeed, the Cochrane Collaboration recommends that tests for funnel plot asymmetry should be used only when there are at least 10 studies included in the meta‐analysis, because when there are fewer studies, the power of the tests is too low to distinguish chance from real asymmetry. But we did not detect any asymmetry, hence, the chance bias does not apply here. Furthermore, Tang and Liu recommend at least six studies for studying publication bias; we included nine [3].

Fifth, the authors would have preferred more in‐depth information about the Bayesian approach and its findings. We used the Bayesian approach to provide a more robust estimate, also given the small number of studies. The similarity of effect estimates does not speak against the approach as such. We agree that the methods are novel to some readers, yet we provide all necessary steps and references in the paper for an interested reader to replicate the procedure in this or other contexts.

Sixth, the authors suggest to use the posterior predictive probability to assess the performance of the prior. We disagree with this view. In the paper referred to by the authors, Lewis et al. stressed the fact that their conditional predictive ordinates approach [4], compared to methods described by Gelfand and Ghosh [5], only evaluates the posterior predictive probability density but does not simulate from it, and both methods relied on applying posterior predictive distribution. Also, in our sensitivity analysis, we checked the prior predictive cumulative distribution function (CDF, as stated in Figure S5 of the Supporting Information of [1]). The CDF is a probability, and the probability density function (PDF) is the derivative of the CDF, and the predictive distribution function is a probability as well. Also, in our view, prior predictive distribution can be understood as a concept in two ways: a PDF in case of a continuous random variable and a probability mass function for discrete values of the random variable. We thus disagree with the authors’ view based on Lewis’ statement. We agree with the view that Bayes factors are useful for guiding an evolutional model‐building process, as stated in the paper from Kass and Raftery [6]. However, it is not the only method for model selection. As evaluated by Lynch and Western [7], the posterior predictive distribution can be compared to the observed data to assess model fit, and the posterior predictive distribution provides a useful set of statistics for assessing model fit.

Finally, the authors would like to see more elaboration on the inferential procedure presented in the manuscript. We agree that Bayesian methods commonly are computationally more demanding than other methods, usually, these require the determination of high‐dimensional integrals, in most cases, with Markov chain Monte Carlo (MCMC) methods. However, in the present case of random‐effects meta‐analysis, where only two unknown parameters are to be inferred, computations may be simplified by utilizing numerical integration or importance resampling [8]. According to Röver and Friede, computations may be done partly analytically and partly numerically, offerring another approach to simplify calculations via the DIRECT algorithm, which is implemented in the bayesmeta R package [9, 10]. Hence, using the bayesmeta R package, direct access to quasi‐analytical posterior distributions can be provided, without having to worry about setup, diagnosis or post‐processing of MCMC algorithms. Also, inference based on MCMC output always contains a certain noise component due to the finite number of samples, which may sometimes constitute a nuisance. Use of the bayesmeta package instantly provides accurate posterior summary figures analogous to output familiar from frequentist meta‐analysis output [10]. Posterior distributions may be accessed in quasi‐analytical form, and advanced methods, for example for prediction or shrinkage estimation, are also provided. Computations are fast and reproducible, allowing for quick sensitivity checks and facilitating larger‐scale simulations. Bayesmeta implementation yields consistent results through calibration checks [10]. Using bayesmeta in R, we thus believe these aspects are not relevant, also given the fact that this is not a methods paper.

## COMPETING INTERESTS

The authors have no competing interests to report.

## AUTHORS’ CONTRIBUTIONS

HW and KJ equally contributed to drafting the response and both have read and approved the final manuscript.
